# Descemet’s membrane endothelial keratoplasty for pseudoexfoliation syndrome: a case series

**DOI:** 10.1186/s12886-019-1130-1

**Published:** 2019-05-28

**Authors:** Saho Tase, Toshiki Shimizu, Takahiko Hayashi, Hitoshi Tabuchi, Koji Niimi, Nobuhisa Mizuki, Naoko Kato

**Affiliations:** 1 Niimi Eye Institute, Hyogo, Japan; 2 Department of Ophthalmology, Yokohama City University Hospital, Kanagawa, Japan; 3 Department of Ophthalmology, Yokohama Minami Kyosai Hospital, 1-21-1, Mutsuura Higashi, Yokohama, Kanagawa 236-0037 Japan; 4 Department of Ophthalmology, Tsukazaki Hospital, Hyogo, Japan; 5 Department of Ophthalmology, Saitama Medical University, Saitama, Japan

**Keywords:** Descemet’s membrane endothelial keratoplasty, Pseudoexfoliation syndrome, Bullous keratopathy, Endothelial keratoplasty

## Abstract

**Background:**

To evaluate the clinical outcomes and features of Descemet’s membrane endothelial keratoplasty (DMEK) for eyes with pseudoexfoliation syndrome (PEX).

**Methods:**

In this retrospective study, 37 DMEK cases were reviewed from available medical records. Patients who exhibited endothelial dysfunction derived from PEX or Fuchs endothelial corneal dystrophy (FECD) and successfully underwent cataract surgery about four weeks before DMEK were enrolled. The best spectacle-corrected visual acuity (BSCVA), central corneal thickness (CCT), endothelial cell density (ECD), and incidence of intra-operative/post-operative complications of DMEK were analyzed.

**Results:**

This study included 14 eyes of 14 patients (PEX: *n* = 6, FECD: *n* = 8). There was no primary graft failure. In the PEX group, BSCVA improved from 0.67 ± 0.28 at the preoperative point to 0.43 ± 0.14 at 1 month, 0.27 ± 0.10 at 3 months, and 0.19 ± 0.08 at 6 months after DMEK. The donor corneal ECD was 2704 ± 225 cells/mm^2^ at the preoperative point and decreased to 1691 ± 498 cells/mm^2^ at 1 month, 1425 ± 366 cells/mm^2^ at 3 months, and 1281 ± 340 cells/mm^2^ (52.7 ± 11.7% less than ECD of the donor graft) at 6 months after DMEK. None of the patients required rebubbling. When compared with the FECD group, no statistical difference was observed in CCT (*p* = 0.821); BSCVA (*p* = 0.001) and the reduction rate of ECD (*p* = 0.010) were relatively worse.

**Conclusions:**

DMEK is effective for the treatment of endothelial dysfunction due to PEX.

**Electronic supplementary material:**

The online version of this article (10.1186/s12886-019-1130-1) contains supplementary material, which is available to authorized users.

## Background

Corneal transplantation is a common procedure. Well over 100,000 cases are performed annually worldwide. About half of all corneal transplantations involve endothelial keratoplasty, which replaces the corneal endothelium with a monolayer of cells. Descemet membrane endothelial keratoplasty (DMEK) is a corneal endothelial keratoplasty newly introduced by Melles et al. that allows for a faster recovery of visual acuity, fewer higher-order aberrations, and lower immunological rejection rates compared to conventional penetrating keratoplasty such as Descemet’s stripping automated endothelial keratoplasty (DSAEK) [1–4].

With the worldwide increase in number of DMEK surgeries, many papers regarding DMEK for Fuchs endothelial corneal dystrophy (FECD) have been published. However, other causes of corneal endothelial dysfunction, such as complications from cataract surgery (pseudophakic bullous keratopathy) and endotheliopathy in pseudoexfoliation syndrome (PEX), are poorly understood [5].

PEX is a genetically determined, age-related, and environmentally influenced disorder characterized by anomalous production and accumulation of abnormal fibrillar extracellular aggregates on anterior segment structures, most notably on the lens capsule and pupillary border of the iris [6–8]. The exfoliative material is often expressed as grey-white and dandruff-like, but its origin is still obscure [9]. The material is observed in multiple organs such as the heart, lung, liver, kidney, cerebral meninges and blood vessels [10, 11]. It is also observed in ocular structures such as the anterior capsule, iris, lens zonule, trabecular meshwork and corneal endothelium. It is the leading cause of glaucoma, cataracts, and bullous keratopathy (BK) [12–14].

Evidence has accumulated reporting the morphological alterations in almost all cell layers of the cornea in eyes with PEX. Eyes with PEX have been documented to have deposition of hyper reflective material on the endothelium, which is presumed to be PEX material, and to have significantly lower cell densities in the basal epithelium, anterior and posterior stromatolites, and endothelium compared to controls [15]. PEX can lead to corneal endothelial cell decompensation, which can result in severe BK, requiring keratoplasty [13].

To our knowledge, this is the first paper to focus on keratoplasty for PEX. Here we describe a case series in which we conduct DMEK for BK derived from PEX and compare the result with that derived from FECD.

## Methods

### Patients and examinations

We complied all ethical principles within the Declaration on Helsinki, and we were approved for the surgical maneuvers and evaluation protocols used in this retrospective study by the Institutional Review Board of Yokohama Minami Kyosai Hospital (Approval no. YKH_30_02_08). We obtained informed consent in written style by patients with endothelial dysfunction derived from PEX or FECD and cataract, and they enrolled this study. The diagnosis of PEX keratopathy was confirmed clinically as well as electron microscopy. Eyes in the PEX group had accumulation of exfoliative materials that was characteristic of PEX and didn’t have other findings, such as guttata and history of past complicated cataract surgery, that could cause BK. Between April 1, 2016, and December 31, 2017, at the department of ophthalmology of Yokohama Minami Kyosai Hospital in Kanagawa, Japan, a total of 37 surgeries were applicable to the study and 14 eyes of 14 patients (6 males and 8 females) were considered eligible. Six eyes revealed PEX syndrome (PEX group), and the other 8 eyes revealed FECD (FECD group).

We performed tests preoperatively and up to 6 months after DMEK. The inspection items were standard ophthalmic examinations, best spectacle-corrected visual acuity (BSCVA), central corneal thickness (CCT), and corneal endothelial cell density (ECD). We also checked corneal endothelial characteristics, graft adaptation and complications after DMEK. CCT was measured using anterior segment optical coherence tomography (AS-OCT, SS1000, Tomey, Aichi, Japan). Preoperative ECDs were derived from the donor eye bank records and postoperative ECDs were measured with the aid of specular microscope (FA3509, Konan Medical, Hyogo, Japan). A form of endothelial cells was measured with the specular microscope, graft adaptation was measured with slit-lamp microscopy and AS-OCT, and clinical/subclinical cystoid macular edema (CME) was measured with spectral-domain OCT (RS 3000, Nidek, Aichi, Japan). CME was confirmed as the presence of intraretinal fluid spaces seen in the fovea region using spectral-domain OCT.

### Cataract surgery

Cataract surgery was scheduled about 4 weeks before DMEK. It was performed under sub-Tenon anesthesia. The pupil was preoperatively treated with a mydriatic agent (0.5% tropicamide and phenylephrine hydrochloride; Mydrin-P; Santen, Japan) to achieve mydriasis. Maximum pre-operative pupil dilation was noted. Phacoemulsification was performed, and the foldable intraocular lens (IOL) was placed in the bag. Five PEX-syndrome patients who needed transscleral-sutured IOL implantation due to zonular dialysis were excluded from this study.

### Surgical procedure of DMEK

A punch was placed on the endothelial surface of the donor disc to indent a circle 7.75, 8.0, or 8.25 mm in diameter. The donor grafts were peeled after staining with 0.1% Brilliant Blue G (BBG) 250 (BBG; Sigma-Aldrich, St. Louis, MO, USA) (1.0 mg/mL). 1.0- and 1.5-mm-diameter dermatological biopsy punches (Kai Industries, Seki, Japan) were used to place asymmetric marks on the edges of the grafts to indicate graft orientation [16]. Donor grafts were cut using the donor punch, stained with 0.1% BBG for 1 min, placed in a balanced salt solution (BSS) (BSS-plus; Alcon, Osaka, Japan) for about 30 min, and used for insertion [17].

All surgeries were performed under retrobulbar block and Nadbath facial nerve block. Two paracenteses and a 2.8-mm upper corneal or corneoscleral tunnel were made for the recipient cornea. Peripheral iridotomy was performed at the 6-o’clock position using a 25-gauge vitreous cutter to prevent the occurrence of a postoperative pupillary block. After the central recipient descemetorhexis under air, the donor membrane graft was inserted into the anterior chamber using an IOL injector (model WJ-60 M; Santen Pharmaceuticals, Osaka, Japan) [5].

The inserted graft was unfolded via a no-touch technique with shallowing of the anterior chamber [18]. After confirmation of the correct orientation of the graft, the anterior chamber was filled with air and partially replaced with BSS 15 min later. Finally, 0.4 mg of betamethasone (Rinderon; Shionogi, Osaka, Japan) was subconjunctivally injected and 1.5% (w/v) levofloxacin eye drops (Cravit; Santen Pharmaceuticals) was administered.

Postoperatively, 1.5% (w/v) levofloxacin (Cravit), 0.1% (w/v) betamethasone sodium phosphate (Sanbetasone; Santen Pharmaceuticals), and 2% (w/v) rebamipide ophthalmic solution (Mucosta; Otsuka, Tokyo, Japan) were prescribed four times daily for 3 months and tapered thereafter.

### Statistical analysis

Male/female and right/left ratios were compared using the χ^2^ test. The paired *t*-test was used to compare preoperative and postoperative values and the unpaired *t*-test was used to compare the PEX group and the FECD group. Moreover, multiple regression analysis was performed after the age adjustment. All analyses were performed using JMP 13.2.0 (SAS institute inc., Cary NC, USA). A *P*-value of< 0.05 was considered to be statistically significant.

## Results

### Patients

The preoperative patient profiles are summarized in Table 1. As shown in Additional file 1: Figure S1, even in PEX patients with severe corneal edema, the cornea edema disappeared and a completely clear cornea was obtained after cataract surgery and DMEK. The mean age of the PEX group was 79.7 ± 5.1 (from 75 to 85 years old); that of the FECD group was 70.4 ± 8.6 (from 55 to 81 years old). The mean age of the PEX group was significantly higher than that of the FECD group (*p* = 0.037). Preoperative BSCVA and CCT before cataract surgery were not significantly different between the PEX and FECD groups (BSCVA; *p* = 0.492, CCT; *p* = 0.710). Preoperatively, none of them was diagnosed a secondary open angle glaucoma (SOAG) with optic nerve damage. The mean pupil diameter was smaller in the PEX group than in the FECD group (*p* = 0.018) and 3 eyes of the PEX group were used capsule expanders due to their zonular weakness at the cataract surgeries. All cataract surgeries were uneventful.

**Table 1:** Patient characteristics before surgery

	PEX	FECD	P*
Number of eyes	6	8	
Sex (male/female)	2 / 4	3 / 5	0.872*
Age	79.7 ± 5.1	70.4 ± 8.6	0.037^†^
Eye (R/L)	3 / 3	6 / 2	0.334*
BSCVA (LogMAR)	0.67 ± 0.28	0.78 ± 0.29	0.492^†^
CCT before cataract surgery (μm)	657.3 ± 60.8	669.1 ± 54.6	0.710^†^
Pupil diameter (mm)	5.67 ± 1.2	7.44 ± 0.5	0.018^†^
Donor age	68.0 ± 2.8	67.0 ± 4.3	0.638^†^

*χ^2^ test ^†^unpaired *t* test

### Visual acuity

**Fig. 1:**
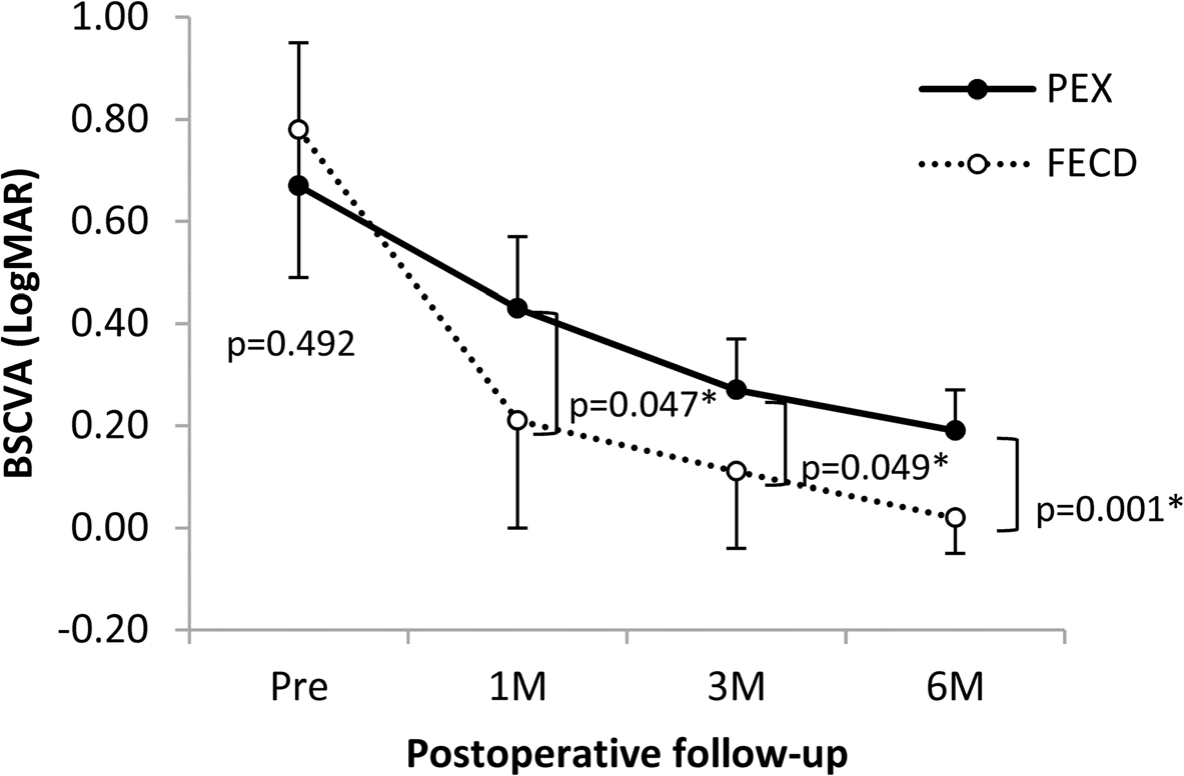
Changes in best spectacle-corrected visual acuity. A statistically significant improvement of BSCVA is obtained in the pseudoexfoliation syndrome (PEX) group except at 1 month (*p* = 0.077 at 1 month, 0.009 at 3 months, 0.003 at 6 months; paired *t*-test). A statistically significant improvement of BSCVA is also obtained in the Fuchs endothelial corneal dystrophy (FECD) group at all examination points (*p* = 0.005 at 1 month, *p* < 0.001 at 3 and 6 months; paired *t*-test). There is no significant difference between the two groups at the preoperative point (*p* = 0.492), whereas the PEX group is significantly worse than the FECD group postoperatively (*p* = 0.047 at 1 month, *p* = 0.049 at 3 months, *p* = 0.001 at 6 months; unpaired *t*-test)

In the PEX group, BSCVA improved from 0.67 ± 0.28 at the preoperative point to 0.43 ± 0.14 at 1 month, 0.27 ± 0.10 at 3 months, and 0.19 ± 0.08 at 6 months after DMEK. In the FECD group, BSCVA improved from 0.78 ± 0.29 at the preoperative point to 0.21 ± 0.21 at 1 month, 0.11 ± 0.15 at 3 months, and 0.017 ± 0.074 at 6 months. A statistically significant improvement of BSCVA was obtained in both groups at all examination points except at 1 month in the PEX group (*p* = 0.077 at 1 month, 0.009 at 3 months, 0.003 at 6 months in the PEX group; *p* = 0.005 at 1 month, *p* < 0.001 at 3 and 6 months in the FECD group, the paired t-test used in both groups). BSCVA was not significantly different between the two groups at the preoperative point (*p* = 0.492). However, the BSCVA of the PEX group became significantly worse than that of the FECD group postoperatively (*p* = 0.047 at 1 month, *p* = 0.049 at 3 months, *p* = 0.001 at 6 months, respectively) (Fig. 1).

### Central corneal thickness

**Fig. 2:**
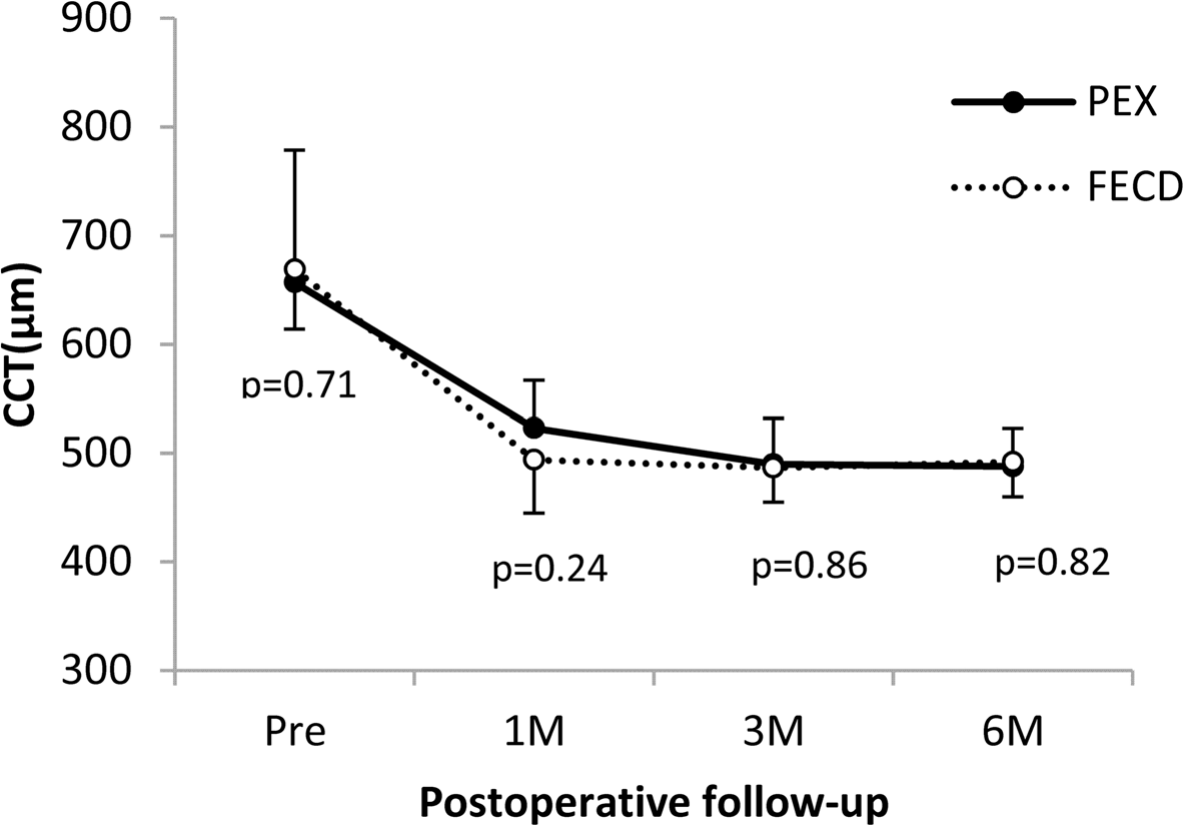
Changes in central corneal thickness. A statistically significant improvement of central corneal thickness (CCT) is obtained in the pseudoexfoliation syndrome (PEX) group at all examination points (*p* = 0.002 at 1 month, 0.002 at 3 months, *p* < 0.001 at 6 months; paired *t*-test). A statistically significant improvement of CCT is also obtained in the Fuchs endothelial dystrophy (FECD) group at all examination points (*p* = 0.001 at 1 month, *p* < 0.001 at 3 and 6 months; paired *t*-test). There is no significant difference between the two groups at all examination points (*p* = 0.71 preoperatively, 0.24 at 1 month, 0.86 at 3 months, 0.82 at 6 months; unpaired *t*-test)

In the PEX group, CCT changed from 657.3 ± 61.1 μm at the preoperative point to 523.2 ± 34.6 μm at 1 month, 489.7 ± 32.5 μm at 3 months, and 488.3 ± 30.1 μm at 6 months after DMEK. In the FECD group, CCT changed from 669.1 ± 54.6 μm at the preoperative point to 494.4 ± 48.6 μm at 1 month, 486.6 ± 31.8 μm at 3 months, and 492.3 ± 32.3 μm at 6 months. A statistically significant improvement of CCT was obtained in both groups at all examination points (*p* = 0.002 at 1 month, 0.002 at 3 months, *p* < 0.001 at 6 months in the PEX group; *p* = 0.001 at 1 month, *p* < 0.001 at 3 and 6 months in the FECD group, the paired t-test used in both groups), and there was no significant difference between the two groups at all examination points (*p* = 0.71 preoperatively, 0.24 at 1 month, 0.86 at 3 months, 0.82 at 6 months; Fig. 2).

### Corneal endothelial cell density

**Fig. 3:**
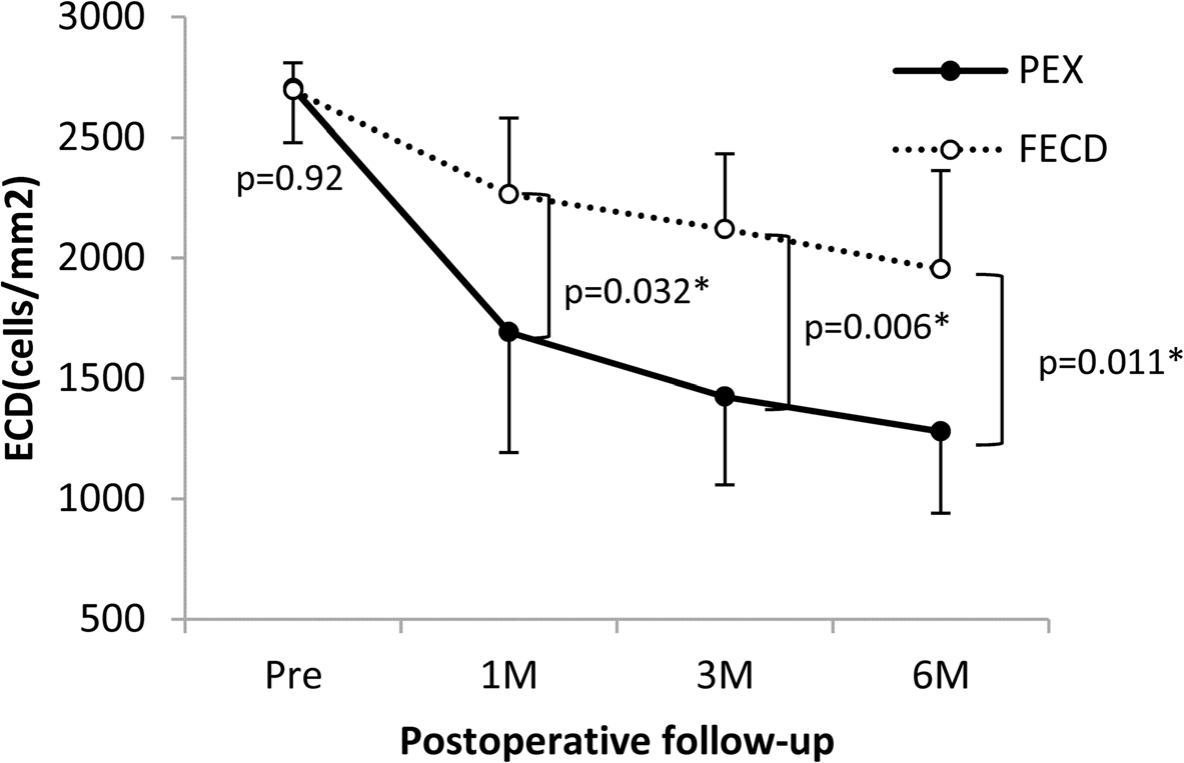
Changes in endothelial cell density. In the pseudoexfoliation syndrome (PEX) group, the donor corneal endothelial cell density (ECD) decreases 2704 ± 225 cells/mm2 at the preoperative point to 1281 ± 340 cells/mm2 at 6 months (52.7 ± 11.7% less than the preoperative value of the donor graft). In the Fuchs endothelial corneal dystrophy (FECD) group, the donor corneal ECD decreases 2694 ± 123 cells/mm2 at the preoperative point to 1954 ± 464 cells/mm2 at 6 months (27.5 ± 17.4% less than the preoperative value of the donor graft). There is no significant difference between the two groups at the preoperative point (*p* = 0.92), whereas the PEX group is significantly worse than the FECD group postoperatively (*p* = 0.032 at 1 month, *p* = 0.006 at 3 months, *p* = 0.011 at 6 months; unpaired *t*-test)

In the PEX group, the donor corneal ECD was 2704 ± 225 cells/mm^2^ at the preoperative point, and decreased to 1691 ± 498 cells/mm^2^ at 1 month, 1425 ± 366 cells/mm^2^ at 3 months, and 1281 ± 340 cells/mm^2^ (52.7 ± 11.7% less than the ECD of the donor graft) at 6 months after DMEK. In the FECD group, the donor corneal ECD was 2694 ± 123 cells/mm^2^ at the preoperative point, and decreased to 2265 ± 386 cells/mm^2^ at 1 month, 2120 ± 402 cells/mm^2^ at 3 months, and 1954 ± 464 cells/mm^2^ (27.5 ± 17.4% of the donor graft) at 6 months after DMEK. Although there was no significant difference between the two groups at the preoperative point (*p* = 0.92). However, the ECD was significantly less in the PEX group compared to the FECD group postoperatively (*p* = 0.032 at 1 month, *p* = 0.006 at 3 months, *p* = 0.011 at 6 months, respectively; Fig. 3).

### Corneal endothelial characteristics

In the PEX group, coefficient of variation (CV) in cell area was 27.2 ± 7.0% at 6 months after DMEK. In the FECD group CV was 33.1 ± 2.6% at 6 months after DMEK. There was no significant difference between the two groups (*p* = 0.12). In the PEX group, cell hexagonality (HEX) was 46.3 ± 13.5% at 6 months after DMEK. In the FECD group HEX was 55.9 ± 9.2% at 6 months after DMEK. There was no significant difference between the two groups (*p* = 0.21).

### Complications after DMEK

None of the eyes showed intraoperative complications, and none revealed primary graft failure. Four eyes (50%) of the FECD group required rebubbling for partial detachment. CME was present in one eye (20%) of the PEX group and one eye (12.5%) of the FECD group. In all affected eyes, the CME resolved within 6 months after the surgery with topical 0.1% (w/v) bromfenac eye drops (Bronuck; Senju Pharmaceuticals) and sub-Tenon injection of 40 mg/mL triamcinolone acetonide (Kenacort A; Bristol-Myers Squibb). None of the eyes revealed postoperative intraocular pressure elevation or exhibited glaucoma.

## Discussion

The current study indicates that DMEK surgery can successfully be performed for eyes with PEX syndrome. Postoperative BSCVA and CCT were significantly improved in both the PEX and FECD groups, even though the final BSCVA was significantly worse and the final ECD was significantly less in the PEX group compared to the FECD group. To the best of our knowledge, this is the first study that focused on the outcomes of DMEK for BK due to PEX and FECD.

Reports have confirmed that the BSCVA of eyes that have undergone DMEK show rapid and sufficient improvement in the early postoperative period. Singh et al. reported that BSCVA was 0.161 ± 0.129 6 months after DMEK and 0.293 ± 0.153 6 months after DSAEK. In the present study, BSCVA 6 months after DMEK in the PEX group (0.193 ± 0.081) was comparable to previously reported results of DMEK and superior to those of DSAEK [19]. On the other hand, BSCVA in the PEX group was inferior to that of the FECD group throughout the 6-month postoperative observation period. And Singh et al. also reported that ECD loss post 6 months was 31% after DMEK. In the present study, ECD loss post 6 months after DMEK in the FECD group (27.5 ± 17.4%) was comparable to the previous results, but significantly worse in the PEX group (52.7 ± 11.7%) [19].

One of the cause is that the mean age of the PEX group was greater than that of the FECD group in the current study. The prevalence of PEX increases progressively with age, and the diagnosis of PEX has rarely been made in individuals younger than 50 [20, 21]. On the other hand, the prevalence of FECD does not significantly increase with age [22]. Subclinical dysfunction of the macula, optical nerves, and brain due to increased age may also contribute to the lower BSCVA in the PEX group. However, after we conducted age-adjusted, BSCVA and ECD loss 6 months after DMEK of the PEX group were still inferior to the FECD group.

We speculated three possible causes for the relatively deteriorated postoperative BSCVA in the PEX group: PEX material may affect the centering of the IOL, posterior segment structures, and/or cognitive function.

It has been well documented that PEX material deposits on the lens zonule cause zonular weakness and contributes to the dislocation of implanted IOLs. Ostern et al. investigated the long-term positioning of the posterior IOL following cataract surgery in eyes with and without PEX and reported that IOLs within the capsular bag were more prone to decentration in eyes with PEX [23]. The dislocated IOL could lead to more higher-order aberration, resulting in the decreased BSCVA.

PEX material has also been reported to accumulate on posterior segment structures such as the choroid and optic nerve. In eyes with PEX, choroidal thinning related to increase vascular resistance and reduce blood flow has been reported [24]. The optic disc area has also been reported as being smaller than controls, both with and without glaucoma [25].

PEX material deposits have also been reported on the cerebral meninges. Magnetic resonance imaging (MRI) of PEX patients with or without glaucoma showed a higher prevalence of white matter hyperintensities than controls [26]. Chronic cerebrovascular disease including senile dementia, cerebral atrophy and cerebral ischemia is reportedly more common in patients with PEX than patients with primary open-angle glaucoma (POAG) [27]. These changes to the posterior structures of the visual pathway may deteriorate BSCVA in patients with PEX.

As is the case with BSCVA, ECD loss post 6 months was also worse in the PEX group. The suggested causes of endotheliopathy include penetration of PEX material towards the Descemet’s membrane [28, 29] and changes in the blood-aqueous barrier [30, 31]. PEX material breaks the hexagonal connections of the endothelial layer and promotes apoptosis. The breakdown of the blood-aqueous barrier caused by PEX iridopathy may have an impact on postoperative ECD. It has been reported that preoperative cytokine levels are associated with ECD loss after DSAEK [32]. Elevated cytokine levels, including pro-inflammatory cytokines and fibrogenic growth factors in the aqueous humor in the PEX group, may facilitate the apoptosis of endothelial cells.

In conclusion, DMEK is effective for the treatment of endothelial dysfunction caused by PEX and FECD. Even though the postoperative BSCVA and ECD were slightly inferior in the eyes with PEX, DMEK provides advantages when compared with other transplant methods, such as DSAEK and penetrating keratoplasty. Future studies involving a larger number of eyes will elucidate the association between PEX and its effect on DMEK.

## Additional file

Additional file 1: **Figure S1.** Before Descemet’s membrane endothelial keratoplasty (DMEK) (**A**) and after DMEK (**B**) in the PEX group. Corneal transparency remarkably improves after phacoemulsification and DMEK. Despite impressive improvement of the corneal edema, the PEX materials are clearly detectable on the iris before and after DMEK (Arrows). (PDF 150 kb)

## Data Availability

The datasets used and/or analyzed during the current study are available from the corresponding author on reasonable request.

## Abbreviations

BSCVA: Best spherical corrected visual acuity
CCT: Central corneal thickness
FECD: Fuchs endothelial corneal dystorophy
LogMAR: Logarithm of the minimum angle of resolution
PEX: Pseudoexfoliation syndrome
