# Supplementary figures and images for: Descemet’s membrane endothelial keratoplasty for pseudoexfoliation syndrome: a case series
Source: BMC Ophthalmol. 2019 May 28;19:119. doi: 10.1186/s12886-019-1130-1 (PMC6537358; doi:10.1186/s12886-019-1130-1)

**Supplemental Figure 1**

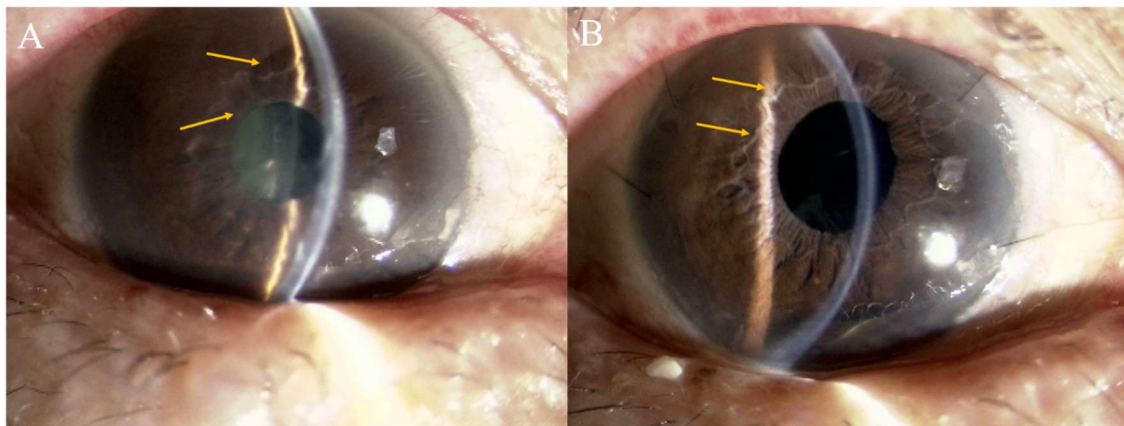

Supplement: Supplementary file 1 — Figure S1. Before Descemet’s membrane endothelial keratoplasty (DMEK) (A) and after DMEK (B) in the PEX group. Corneal transparency remarkably improves after phacoemulsification and DMEK. Despite impressive improvement of the corneal edema, the PEX materials are clearly detectable on the iris before and after DMEK (Arrows). (PDF 150 kb) [file 12886_2019_1130_MOESM1_ESM.pdf]
